# Evaluation of Peripheral Immune Activation in Amyotrophic Lateral Sclerosis

**DOI:** 10.3389/fneur.2021.628710

**Published:** 2021-06-24

**Authors:** Mengli Wang, Zhen Liu, Juan Du, Yanchun Yuan, Bin Jiao, Xuewei Zhang, Xuan Hou, Lu Shen, Jifeng Guo, Hong Jiang, Kun Xia, Jianguang Tang, Ruxu Zhang, Beisha Tang, Junling Wang

**Affiliations:** ^1^Department of Neurology, The Third Xiangya Hospital, Central South University, Changsha, China; ^2^Department of Neurology, Xiangya Hospital, Central South University, Changsha, China; ^3^Health Management Center, Xiangya Hospital, Central South University, Changsha, China; ^4^Laboratory of Medical Genetics, Central South University, Changsha, China; ^5^Key Laboratory of Hunan Province in Neurodegenerative Disorders, Central South University, Changsha, China; ^6^National Clinical Research Center for Geriatric Diseases, Xiangya Hospital, Central South University, Changsha, China; ^7^Department of Neurology, The Second Xiangya Hospital, Central South University, Changsha, China

**Keywords:** immunoglobulin, complement, amyotrophic lateral sclerosis, neurodegenerative diseases, peripheral immune activation

## Abstract

Accumulating evidence has revealed that immunity plays an important role in amyotrophic lateral sclerosis (ALS) progression. However, the results regarding the serum levels of immunoglobulin and complement are inconsistent in patients with ALS. Although immune dysfunctions have also been reported in patients with other neurodegenerative diseases, few studies have explored whether immune dysfunction in ALS is similar to that in other neurodegenerative diseases. Therefore, we performed this study to address these gaps. In the present study, serum levels of immunoglobulin and complement were measured in 245 patients with ALS, 65 patients with multiple system atrophy (MSA), 60 patients with Parkinson's disease (PD), and 82 healthy controls (HCs). Multiple comparisons revealed that no significant differences existed between patients with ALS and other neurodegenerative diseases in immunoglobulin and complement levels. Meta-analysis based on data from our cohort and eight published articles was performed to evaluate the serum immunoglobulin and complement between patients with ALS and HCs. The pooled results showed that patients with ALS had higher C4 levels than HCs. In addition, we found that the IgG levels were lower in early-onset ALS patients than in late-onset ALS patients and HCs, and the correlations between age at onset of ALS and IgG or IgA levels were significant positive. In conclusion, our data supplement existing literature on understanding the role of peripheral immunity in ALS.

## Introduction

Amyotrophic lateral sclerosis (ALS) is a devastating neurodegenerative disease characterized by the progressive degeneration of upper and lower motor neurons at the motor cortex, bulbar, and spinal levels, leading to muscle weakness, progressive paralysis, and respiratory failure and death within 3–5 years after disease onset (1). Whilst the etiology and pathology of ALS remains unclear, current evidence suggests that oxidative stress, glutamatergic toxicity, ribonucleic acid (RNA) processing dysfunction, abnormal protein aggregation, mitochondrial dysfunction, axonal transport dysfunction, neuroinflammation, and immunomodulation might play important roles in the pathogenesis of ALS (2, 3).

Recently, evidence indicating the significant contribution of inflammatory and immune responses to the progression of ALS has emerged (4). The activation of astrocytes and microglia and the infiltration of T cells and monocytes have been observed in the central nervous system (CNS) of patients with ALS and animal models (5–8). Immunoglobulin deposits were observed in motor neurons from the spinal cord of patients with ALS (9), and upregulated complements were found in the cerebrospinal fluid (CSF), spinal cord, and motor cortex of patients with ALS and transgenic mice (10–12). In addition to the dysregulation of immune responses in the CNS, immune aberrations within the peripheral nervous system have been reported in patients with ALS. The levels of circulating chemokines and cytokines, such as IL-7, IL−6, and TNF-alpha and its soluble receptors, TNF-R1 and TNF-R2, were found to be increased in patients with ALS (13–15). Abnormality of certain complement factors in complement system, a central component of the innate immune system, which activated by three different pathways [the lectin (LP), the classical (CP), and the alternative pathway (AP)] also were found in the circulation of patients with ALS (16, 17). Increased levels of complement C3 and C5a, C reactive protein (CRP), and erythrocyte sedimentation rate (ESR) as well as T cell abnormalities and immunoglobulin alterations were found in the blood of patients with ALS (16, 18–22). However, some results regarding serum immunoglobulin and complement are inconsistent. Some studies demonstrated significantly higher IgG levels, not IgM or IgA levels, in patients with ALS than in controls (23, 24). Other studies reported normal serum levels of IgG, IgA, and IgM (25, 26), or increased IgM levels and normal IgG level at the early stage of ALS, with a decrease in IgG levels and serum IgM level normalization with disease progression (27). Additionally, although there is strong evidence of complement upregulation in ALS, normal C3 and C4 levels were also observed in patients with ALS (25, 28), and the specific genetic deletion of C3 and C4 in ALS mouse models did not show any beneficial effects on the disease progression (12, 29).

Neuroinflammation and immunomodulation have been hypothesized to play roles in the pathogenesis of ALS as well as other neurodegenerative diseases, such as Alzheimer's disease (AD), Parkinson's disease (PD), and multiple system atrophy (MSA) (30–37). Important differences in the immunological processes occurring in ALS, PD, and AD involving microglial function have been reported. Furthermore, although the contribution of adaptive immune cells to AD seems to be modest, T cells can influence microglial phenotypes and induce neuroprotection in PD, and especially in ALS models (34). In addition, an increasing number of studies on peripheral immune component levels in patients with other neurodegenerative diseases, such as AD and PD, have been conducted (38, 39); however, few studies have explored whether the contribution of peripheral immune components is similar among patients with ALS and other neurodegenerative diseases.

To evaluate these conflicting findings, resolve any discrepancies, and explore the relationships between ALS clinical characteristics and peripheral immune components as well as determine whether ALS shares similar peripheral immune abnormalities with other neurodegenerative diseases, the current study was designed: (1) to detect differences, if any, in the serum levels of IgG, IgM, IgA, and complement C3 and C4 among patients with ALS, patients with other neurodegenerative diseases, and healthy controls (HCs); (2) to systematically review the results of previous studies as well as our cohort using the meta-analysis methodology; and (3) to compare the serum immune component levels between different ALS patient groups established by age at onset, site at onset, disease duration, or disease severity, and analyse the correlations of serum immune components with age at onset, disease duration, the revised ALS functional rating scale (ALSFRS-R) score, and disease progression.

## Materials and Methods

### Subjects

A total of 245 patients with ALS in the Department of Neurology in Xiangya Hospital at Central South University (CSU) in China from April 2013 to April 2019 were enrolled in the study. All patients were diagnosed with definite, probable, or probable-laboratory-supported ALS by at least two neurologists according to the revised El Escorial criteria 2015 (40). Their demographic features and clinical data, including sex, age, site at onset, age at onset, disease duration, and the ALSFRS-R score, were collected. All data were collected when the blood tests were performed.

Two control groups were included in the study. A total of 82 sex- and age-matched healthy individuals without any neurological diseases were recruited from the Health Management Center in Xiangya Hospital at CSU as HCs. In addition, 65 sex- and age-matched patients with MSA and 60 matched patients with PD recruited from the same location as the ALS patients were enrolled as neurological disease controls. All patients with PD were diagnosed according to the Movement Disorder Society (MDS) clinical diagnostic criteria (41). All patients with MSA were diagnosed according to the current consensus criteria established by Gilman and colleagues (42).

Subjects with systemic inflammation, monoclonal gammopathy, non-malignant endocrine abnormalities, neoplastic disorders, auto-antibodies, and infection, or those who had a history of using anti-inflammatory drugs, acetylsalicylic acid, steroids, and statins within the 2 months before enrolment were excluded from this study. This study was approved by the Ethics Committee of Xiangya Hospital at CSU in China, and all subjects included provided written informed consent prior to participation.

### Biochemical Methods

Ten milliliters of peripheral venous blood samples were collected from all subjects in accordance with the study protocols. Then, each blood sample that was collected was sent to the Clinical Laboratory at Xiangya Hospital, and the levels of immunoglobulin and complement were measured using Luminex rate nephelometry (Beckman Coulter IMMAGE 800). All procedures were performed in the accordance with the instructions of manufacturer.

### Statistical Analysis

All analyses were performed using SPSS 22.0 (SPSS, Chicago, IL, USA) and GraphPad Prism 5.03(®GraphPad Software, Inc.). Differences with *p* < 0.05 were considered statistically significant, and multiple comparisons were performed with Bonferroni correction. All continuous data, including those of age, disease duration, age at onset, the ALSFRS-R scores, and the levels of IgG, IgA, IgM, C3, and C4 are presented as means ± standard deviations. The Chi-square test was used to analyse the differences in the sex distributions among different groups. A one-way analysis of variance (ANOVA) was used to analyse the differences in age, disease duration, age at onset, the ALSFRS-R scores, and the levels of IgG, IgA, IgM, C3, and C4 among more than two groups; then, Dunn's multiple comparison test was performed. The correlations between serum immune variables and ALS clinical characteristics, including age at onset, disease duration, and the ALSFRS-R score, were analyzed using Spearman's correlation analysis.

### Meta-Analysis

The methods of search strategy, inclusion and exclusion criteria, outcome measures, and statistical analysis followed the Preferred Reporting Items for Systematic Reviews and Meta-Analyses (PRISMA) statement (43). The Embase, Pubmed, and the Cochrane Library primary databases were searched using the following search terms: “amyotrophic lateral sclerosis” OR “ALS” OR “motor neuron disease” OR “MND” combined with the terms “immunoglobulin” OR “IgG” OR “IgM” OR “IgA” OR “complement” OR “C3” OR “C4.” The language of the articles was restricted to English, and all searches were performed prior to April 6, 2020.

All the articles were carefully read and evaluated. Studies were included if they met the following criteria: (1) observational studies (including case-control studies or cohorts) that evaluated the serum levels of immunoglobulin or complement between patients with ALS and subjects without neurological diseases; (2) the ALS diagnostic criteria were clearly stated; and (3) met at least six points of the Newcastle-Ottawa Scale criteria (NOS) (44), which was used to evaluate the methodological quality of case-control and cohort studies. Review articles, editorials, commentaries, case reports, animal experiments, hypothesis papers, letters that reported no new data, meta-analyses, and abstracts were excluded.

Two investigators independently selected studies and extracted data according to the inclusion criteria, and a third researcher was asked to resolve disputes. The following data were extracted: the first author, publication year, country, sample size, mean age of the study group, mean and standard derivations of the serum levels of immunoglobulin and complement, methods used to measure the levels of immunoglobulin or complement measurement.

In the meta-analysis, the primary summary measure was the standardized mean difference (SMD) in serum immunoglobulin and complement levels between patients with ALS and control subjects. The SMDs with 95% CIs were calculated using a random-effects model and presented in a forest plot for each immunity-related variable. The level of heterogeneity across studies was evaluated using the *I*^2^ statistic (45) and Galbraith graph, and sensitivity analysis was performed by study-by-study exclusion. Publication bias was assessed using funnel plots, as well as Egger's and Begg's tests. A two-tailed *p* < 0.05 was considered statistically significant. All data analyses were performed using STATA (version 13).

## Results

### Demographic and Clinical Features of Subjects

The demographic and clinical features of the subjects are summarized in Table 1. There were no significant differences between patient groups and the HC group with regard to age and sex. For the ALS group, the average age at onset was 54.56 ± 9.71 years (range 25–73 years), the average disease duration for ALS was 16.20 ± 16.59 months with a mean ALSFRS-R score of 39.20 ± 5.64, and 52 patients (21.2%) had with bulbar-onset symptoms.

**Table 1:** Characteristics of patients with ALS, MSA, and PD and HCs.

	**ALS**	**MSA**	**PD**	**Healthy controls**	***P*-value**
Number	245	65	60	82	
Age (years)	55.87 ± 9.71	58.02 ± 7.95	57.08 ± 10.49	54.98 ± 7.547	0.186
Sex, *n* (%)					0.213
Male	162 (66.1%)	40 (61.5%)	31 (51.7%)	50 (61.0%)	
Female	83 (33.9%)	25 (38.5%)	29 (48.3%)	32 (39.0%)	
Age at onset (years)	54.56 ± 9.71		-	-	
Disease duration (months)	16.20 ± 16.59		-	-	
ALSFRS-R score	39.20 ± 5.64		-	-	
Onset site, *n* (%)					
Spinal	193 (78.8%)		-	-	
Bulbar	52 (21.2%)		-	-	

*Values are showed as mean (± standard deviation) or percentage*.

### Serum Levels of Immunoglobin and Complement in Patients With ALS, MSA, and PD, and HCs

**Figure 1:**
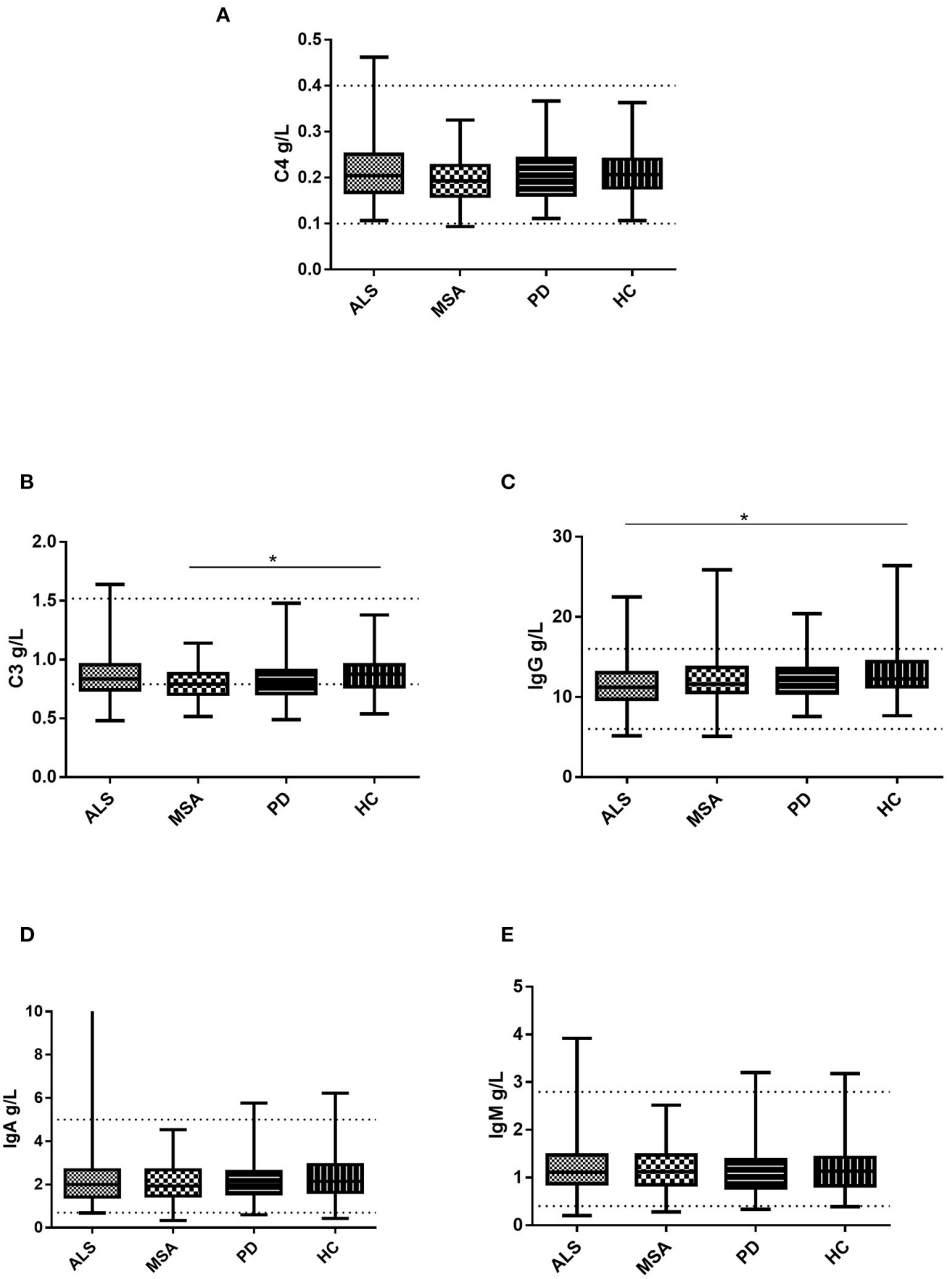
Levels of the serum immunoglobulin and complement in patients with ALS, patients with MSA, patients with PD, and HCs. **(A)** Levels of serum C4 in patients with ALS, patients with MSA, patients with PD, and HCs. **(B)** Levels of serum C3 in patients with ALS, patients with MSA, patients with PD, and HCs. **(C)** Levels of serum IgG in patients with ALS, patients with MSA, patients with PD, and HCs. **(D)** Levels of serum IgA in patients with ALS, patients with MSA, patients with PD, and HCs. **(E)** Levels of serum IgM in patients with ALS, patients with MSA, patients with PD, and HCs. *p < 0.05.

There were no significant differences in the levels of IgA, IgM, or C4 among the patients with ALS, MSA, and PD and HC (Table 2 and Figure 1). The levels of C3 were observed to be lower in the serum of the patients with MSA than in that of the HCs. No significant differences in serum C3 levels were found between the ALS patients and HCs, PD patients and HCs, ALS patients and MSA patients, or ALS patients and MSA patients. The patients with ALS had lower serum IgG levels than did the HCs. No significant differences in serum IgG levels existed between the MSA patients and HCs, PD patients and HCs, ALS patients and MSA patients, or ALS patients and PD patients.

**Table 2:** Levels of serum immunoglobulin and complement in patients with ALS, MSA, and PD and HCs.

	**ALS**	**MSA**	**PD**	**HC**	***p*-value (ALS vs. HC)**	***p*-value (MSA vs. HC)**	***p*-value (PD vs. HC)**	***p*-value (ALS vs. MSA)**	***p*-value (ALS vs. PD)**	***p*-value (MSA vs. PD)**
C4 (g/L)	0.22 ± 0.06	0.20 ± 0.05	0.20 ± 0.05	0.21 ± 0.05	0.744	0.041	0.163	0.017	0.085	0.586
C3 (g/L)	0.85 ± 0.16	0.80 ± 0.14	0.80 ± 0.16	0.87 ± 0.16	0.385	**0.004**	0.010	0.010	0.026	0.813
IgG (g/L)	11.50 ± 2.59	12.20 ± 3.20	12.18 ± 2.64	12.73 ± 2.74	** <0.001**	0.293	0.244	0.067	0.070	0.972
IgA (g/L)	2.14 ± 1.04	2.14 ± 0.97	2.22 ± 0.92	2.32 ± 0.98	0.169	0.273	0.529	0.995	0.597	0.654
IgM (g/L)	1.24 ± 0.59	1.18 ± 0.51	1.16 ± 0.52	1.12 ± 0.48	0.448	0.915	0.720	0.429	0.309	0.817

*Values are shown as the means (± standard deviations). Multiple comparisons were conducted with Bonferroni correction. p <0.009 was considered statistically significant in this analysis. P-value with significance was highlighted with bold*.

### Meta-Analysis Comparing the Serum Levels of Immunoglobulin and Complement Between Patients With ALS and Controls

**Figure 2:**
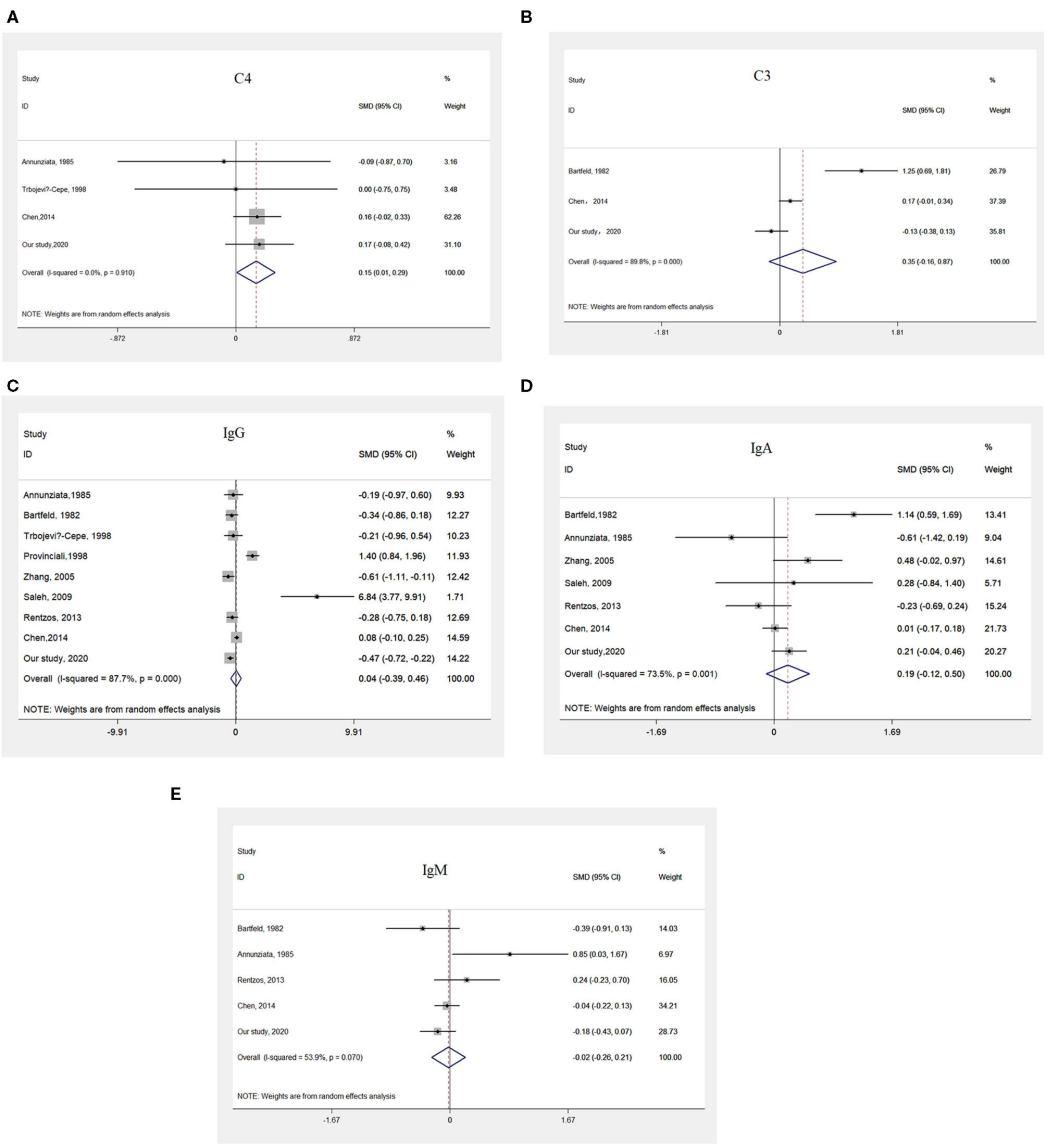
Meta-analysis comparing the serum levels of immunoglobulin and complement between patients with ALS and HCs. **(A)** Pooled results indicating higher serum levels of C4 in patients with ALS than in HCs. **(B)** Pooled results suggesting no differences in the serum levels of C3 between patients with ALS and HCs. **(C)** Pooled results suggesting no difference in the serum levels of IgG between patients with ALS and HCs. **(D)** Pooled results suggesting no difference in the serum levels of IgA between patients with ALS and HCs. **(E)** Pooled results suggesting no difference in the serum levels of IgM between patients with ALS and HCs.

The pooled results from four studies (45, 25, 46; and our study), representing 554 patients with ALS and 327 controls (Supplementary Table 4), suggested that patients with ALS had higher serum C4 levels than did controls (Figure 2A).

The pooled results from three studies (47, 25; and our study), including a total of 572 patients with ALS and 321 controls (Supplementary Table 5), did not show any differences in serum C3 levels between patients with ALS and controls (Figure 2B).

After a systematic search and evaluation were performed, the results of eight published articles (24–27, 46–49) together with our results, representing data from 711 patients with ALS and 477 controls, met the inclusion criteria for a meta-analysis of IgG (Supplementary Table 1). The pooled results suggested there were no differences in serum IgG levels between patients with ALS and controls (Figure 2C).

Five studies(45, 47, 25, 26; and our study), including a total of 621 patients with ALS and 368 controls, were included in the meta-analysis of IgA (Supplementary Table 2), and no significant differences in serum IgA levels between patients with ALS and controls were identified (Figure 2D).

The pooled results from seven studies (45, 25, 26, 24, 27; and our study), including a total of 667 patients with ALS and 401 controls (Supplementary Table 3), showed no significant differences in serum IgM levels between patients with ALS and controls (Figure 2E).

The process of screening studies in the literature, the characteristics of the studies included in the meta-analysis, and the heterogeneity analysis, sensitivity analysis and publication bias results are shown in Supplementary Tables 1–5 and Supplementary Figures 1–4.

### The Relationship Between Serum Immunoglobulin and Complement and Clinical Characteristics of ALS

We compared serum levels of immunoglobulin and complement between different ALS patient groups, including early- and late-onset ALS patient groups (established according to whether the age at onset of ALS was younger or older than 55 years of age), ALS patient groups with short and long disease durations (grouped by whether their disease duration of ALS was longer than 12 months), mild and moderate-to-severe ALS patient groups (grouped according to whether the ALSFRS-R score was >36), ALS patient groups with bulbar onset or spinal onset, and slow and fast progression ALS patient groups (grouped according to whether the (48- score of ALSFRS-R)/disease duration>1).

**Figure 3:**
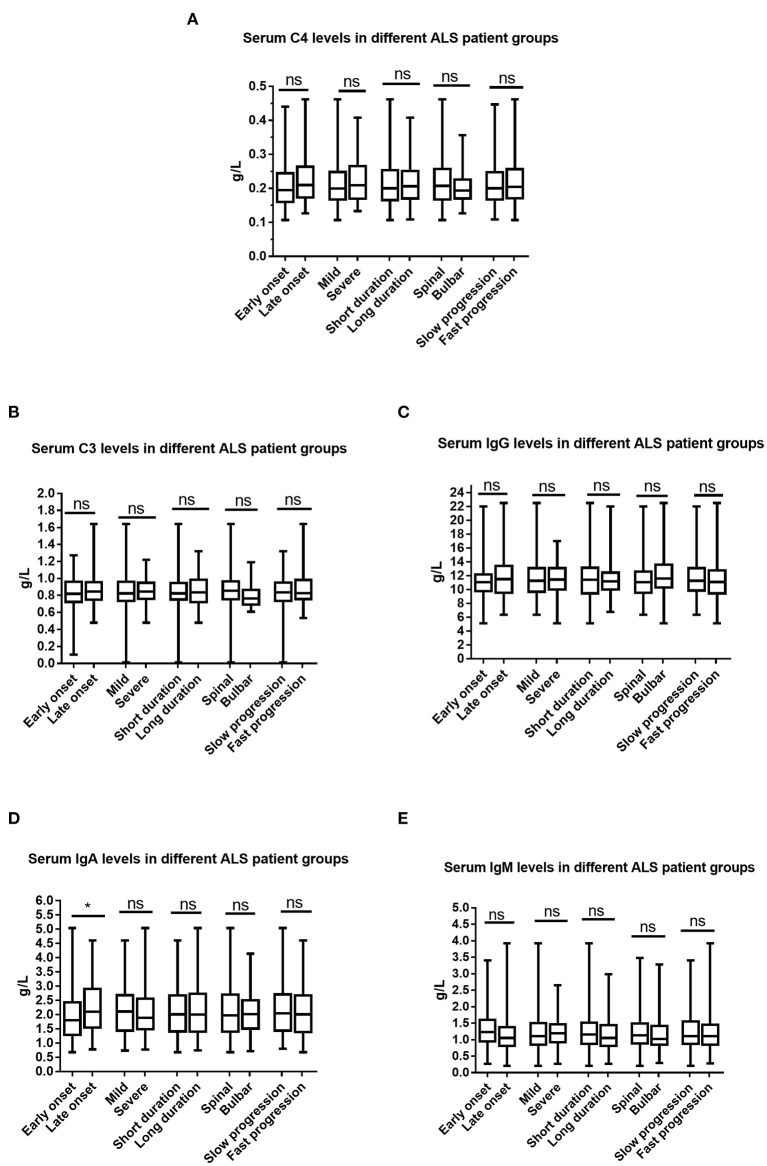
Comparisons of the serum immunoglobulin and complement between different ALS patient groups. **(A)** Serum C4 between different ALS patient groups. **(B)** Serum C3 between different ALS patient groups. **(C)** Serum IgG between different ALS patient groups. **(D)** Serum IgA between different ALS patient groups. **(E)** Serum IgM between different ALS patient groups. Ns denotes no significance, *p < 0.05. Early onset indicated ALS patient group with an age at onset of <55 years old; Late onset indicated ALS patient group with an age at onset of ≥55 years old; Short duration indicated ALS patient group with a disease duration of <12 months, Long duration ALS patient group with a disease duration of ≥12 months; Spinal onset indicated ALS patient group with symptoms at onset involving only the spinal cord; Bulbar onset indicated ALS patient group with symptoms at onset involving the bulbar region; Mild indicated ALS patient group with an ALSFRS-R score of ≥37; Severe indicated ALS patient group with an ALSFRS-R score of <37.

Sex, age, age at onset, disease duration, and the ALSFRS-R score have been adjusted when the ALS patient subgroups were established according to age at onset, site at onset, disease duration, or disease severity. As shown in Figure 3, we only observed differences in serum levels of IgA between patients with early-onset and late-onset ALS.

As shown in Table 3, age at onset was significantly positively correlated with the serum levels of IgA and IgG. No significant correlations were observed between age at onset and IgM, C3, or C4. In addition, no significant correlations were found between the ALSFRS-R score, disease duration, disease progression, and the serum levels of immunoglobulin and complement.

**Table 3:** Correlation analysis between the serum levels of immunoglobulin or complement and clinical characteristics of ALS.

	**Age at onset**		**Disease duration**		**ALSFRS-R score**		**Disease progression**	
	**#rs**	***P*-value**	**rs**	***P*-value**	**rs**	***P*-value**	**rs**	***P*-value**
C4	0.088	0.170	0.025	0.703	−0.059	0.354	0.013	0.845
C3	−0.042	0.508	0.014	0.825	−0.008	0.901	0.036	0.590
IgG	0.130	**0.041**	0.026	0.684	0.001	0.982	−0.071	0.284
IgA	0.158	**0.013**	−0.035	0.591	−0.003	0.963	−0.017	0.284
IgM	−0.091	0.154	−0.083	0.196	0.031	0.632	0.022	0.743

^#^*rs, correlation coefficients. P-value with significance was highlighted with bold*.

In addition, we performed the correlation analysis between age and the levels of C3, C4, IgG, IgA, IgM in healthy controls, no significant correlation was found. And there was no difference in the levels of IgA between healthy controls aged more than 55 age old and healthy controls aged no more than 55 age old.

## Discussion

In the present study, a meta-analysis including our data indicated that the serum levels of IgG, IgA, IgM, and C3 in patients with ALS did not differ significantly from those in HCs, while the serum levels of C4 were higher in ALS patients than in HCs. Although differences in the serum levels of immunoglobulin and complement between patients with ALS and HCs have been reported in several studies, some of the results are conflicting. The discrepancy may be related to the heterogeneity of the patient cohorts and the limited number of patients. One way to address this problem is to combine all available published data in a systematic meta-analysis. To our knowledge, our current study is the first meta-analysis conducted to draw systematic conclusions from these inconsistent results. The pooled results in the present study supported the hypothesis that peripheral complement activation exists in the ALS patients. In addition, the levels of C4d and C4 in the CFS and the glial cells in the vicinity of motor neurons in the spinal cord and motor cortex were observed to be higher in ALS patients than in HCs (37, 50), providing evidence of the complement C4 activation in the CNS of ALS patients. Therefore, the classic complement pathway may be involved in the pathophysiology of ALS. However, where these complement factors originate and what initiates their activation remain unknown.

The activation of complement system is a cascade. The LP is activated when the C2 and C4 are cleaved, then a C3 convertase (C4b2a) is formed. C3 convertase cleaves C3 into C3a and C3b and leads to the formation of a C5 convertase (C4b2a3b), which cleaves the terminal pathway component C5 into C5a and C5b. Then C5b binds C6 and C7 in fluid phase to form C5b-7 complex. The complex attaches to membrane and mediates the formation of the terminal pathway membrane attack complex (C5b-9) which causes direct cytolysis. The anaphylatoxins C3a and C5a trigger proinflammatory signal through binding to C3a receptor (C3aR) and C5a receptor (C5aR), specific G-protein coupled receptors, on innate immune cells. Furthermore, C3a and C5a are potent chemoattractants that attract immune cells to the site of complement activation and promotes phagocytosis of opsonised targets (17). The CP is activated by the pattern recognition molecules C1q in complex with the serine proteases C1r and C1s, and the activation leads to cleavage of C4 and C2, and the formation of a C3 convertase (C4b2a) (51). The CP merge with the LP in the formation of a C3 convertase, and the downstream activation is equivalent. The AP is activated by C3 spontaneous hydrolysis which lead to factor B (FB) binding to C3b (52, 53). FB is then cleaved by Factor D leading to the formation of the AP C3 convertase (C3bBb) which results in the formation of AP C5 convertase (C3bBb3b).

Increased levels of C5a have been described in plasma as well as in the leukocytes of the peripheral blood of patients with ALS (28), and a significant upregulation of ficolin-3 which increase the potential of LP complement activation also been reported (54). Intriguingly, a downregulation of clusterin which is a soluble complement inhibitor of the formation of the transtmembrane C5b-9 was been found. These novel findings further implicate a dysregulated complement system in the pathogenesis of ALS.

Noticeably, immune responses, both innate and adaptive, might highly influence disease progression by shifting from beneficial (neuroprotective) to deleterious (neurotoxic) immune responses. For example, microglia can be activated and differentiate in a pro-inflammatory (Ma) phenotype in response to injury or antigen, and then converse to an anti-inflammatory phenotype (M2) once the inciting event has been dealt with. M2 microglia can secrete anti-inflammatory cytokines as well as neurotrophic factors such as brain-derived neurotrophic factor (BDNF), and insulin-like growth factor-1 (IGF1) which has the capacity to promote growth and differentiation of “neural stem cells” (55), while impairment of GH-IGF system has been shown in ALS (56). Thus, neurodegeneration is facilitated by the lack of neurotrophic growth factors and by the continued production of cytotoxic byproducts of a pro-inflammatory response.

Although patients with MSA tend to have lower serum C3 levels than do HCs, no differences in the serum levels of immunoglobulin and complement were observed between ALS and MSA patients or between ALS and PD patients. In addition, certain shared pathogenic features involving macrophage activation in the blood of patients with ALS and other neurodegenerative diseases have also been reported (27). Therefore, ALS seems to share similar features with other neurodegenerative diseases regarding peripheral immunity activation.

We found that the serum levels of IgA were lower in patients with early-onset ALS than in patients with late-onset ALS, and that the serum levels of IgA and IgG were positively correlated with age at onset. A recent study suggested that Chinese patients with sporadic ALS have a relatively long survival time, probably due to their young age and the small number of bulbar-onset cases (57). Therefore, we hypothesized that ALS patients with lower serum IgA levels may have a longer survival time due to their younger age at onset.

In the present study, no differences in the serum levels of immunoglobulin and complement were observed between mild and moderate-to-severe ALS patients, and no correlations existed between the ALSFRS-R score and the serum levels of immunoglobulin and complement which was consistent with previous results (26). However, Zhang et al. (27) demonstrated that the levels of serum IgM in patients with mild-to-moderate ALS were significantly higher than those in normal controls, but there were no differences between patients with severe ALS and normal controls. Chen et al. (25) found that the levels of IgG were lower in ALS patients with moderate (ALSFRS-R range 25-36) and severe (ALSFRS-R range 0-24) impairment than in those with mild (ALSFRS-R range 37-48) impairment. They also revealed a significantly positive correlation between the ALSFRS-R scores and levels of IgG. The discrepancy may be attributed to the heterogeneity in the patient cohorts.

We observed that serum levels of IgG, IgA, IgM, C4, and C3 did not vary or correlate with disease duration, which was consistent with previous report (26). However, Chen et al. (25) identified a positive correlation between IgG serum levels and disease duration. This inconsistency may be related to the heterogeneity of the patient cohorts.

We did not observe any differences in the serum levels of immunoglobulin or complement between spinal-onset and bulbar-onset ALS patients, which agrees with the observations from a previous study demonstrating that the levels of IgG, IgA, and IgM in serum do not differ significantly in ALS patients with or without bulbar-onset (26). Noticeably, differences in pathology between bulbar-onset and spinal-onset ALS patients have been reported. For example, some bulbar-onset ALS cases presented with atypical pathology such as neurofibrillary tangles and basophilic inclusion, which were not found in the spinal-onset ALS cases (58).

Considering the implication of immune/inflammatory in the pathogenesis of ALS, several therapeutic trials aiming at modulating immunity in ALS have been carried out. To block terminal complement C5 activation and reduce neuroinflammation, the long-acting humanized monoclonal antibody C5 complement inhibitor Ravulizumab-cwvz, which is approved d as a treatment for atypical hemolytic uremic syndrome and paroxysmal nocturnal hemoglobinuria, is now in a phase 3 randomized clinical trial for ALS (59, 60). Additionally, to treat ALS through promoting the growth of nerve tissue and improving neuroprotective function, mesenchymal stem cell (MSC)-neurotrophic factor (NTF) cells have been developed (61). NurOwn is an autologous bone marrow-derived MSC platform that expands and induces the cells to secrete high levels of NTFs (MSC-NTF), and a phase 2 safety and efficacy study randomized 48 patients with an ALSFRS-R of at least 30 have confirmed its efficacy and benefit (62). Now, NurOwn is in the phase 3 trial (ClinicalTrials.gov Identifier: NCT03280056) (59).

In conclusion, this study validated the existence of peripheral immune abnormalities in ALS patients and explored the association between peripheral immunity and the clinical characteristics of ALS. We also demonstrated that ALS may share similar features with PD and MSA regarding peripheral immunity activation. In addition, our data suggest that lower IgA levels might be related to the early onset of ALS. Our study provides additional evidence for researchers on the pathogenesis of ALS and shed light on potential therapeutic interventions aimed at modulating immunity.

## Data Availability Statement

The raw data supporting the conclusions of this article will be made available by the authors, without undue reservation.

## Ethics Statement

The studies involving human participants were reviewed and approved by the Ethics Committee of Xiangya Hospital at Central South University in China. The patients/participants provided their written informed consent to participate in this study.

## Author Contributions

MW conducted data analysis and drafted the paper. ZL, JD, YY, BJ, and XZ contributed to the collection of sample population and blood samples. XH, LS, JG, HJ, KX, JT, RZ, and BT supervised the research. JW designed the experiment, revised the manuscript, and supervised the research. All authors read and approved the final manuscript.

## Conflict of Interest

The authors declare that the research was conducted in the absence of any commercial or financial relationships that could be construed as a potential conflict of interest.
