# Supplementary material for: Evaluation of Peripheral Immune Activation in Amyotrophic Lateral Sclerosis
Source: Front Neurol. 2021 Jun 24;12:628710. doi: 10.3389/fneur.2021.628710 (PMC8264193; doi:10.3389/fneur.2021.628710)
Supplement: Supplementary file 1 [file Table_1.DOC]

**Supplementary Table 1** Studies included in the meta-analysis for evaluating the serum IgG levels between patients with ALS and controls.

| Study | Country | Method of IgG measurement | Cases | | | Control | | | | | NOS score |
| --- | --- | --- | --- | --- | --- | --- | --- | --- | --- | --- | --- |
|  |  |  | n | Mean age (range), years | Mean serum IgG (SD), g/L | n | Mean age (range), years | Mean serum IgG (SD), g/L | Control selection | matching |  |
| Bartfeld,1982 | USA | RID | 43 | U/S | 12.42(±0.69)^#^ | 22 | age range comparable | 12.6(±0.66)^#^ | Spouses and blood donor | GM, AM | 7 |
| Annunziata, 1985 | Italy | RID | 13 | U/S(37-72) | 13.5(±2.99)^#^ | 12 | U/S | 14(±2.24)^#^ | Unaffected ^*^ | U/S | 7 |
| Trbojević-Cepe, 1998 | Croatia | LN | 12 | U/S | 11.4(±2.6) | 16 | U/S | 11.9(±2.3) | Unaffected | U/S | 6 |
| Provinciali, 1998 | Italy | RID | 32 | 59 | 12.17(±3.39)^#^ | 30 | 56 | 8.0(±2.49)^#^ | Unaffected | AM | 7 |
| Zhang, 2005 | USA | ELISA | 38 | 59.3(±13.4) | 7.8(±5.76)^#^ | 28 | 56.0(±15.1) | 11.26(±5.57)^#^ | Normal blood donor | AM | 8 |
| Saleh, 2009 | USA | U/S | 8 | 53.2(±3.1) | 12.52(±0.66)^#^ | 5 | 59.8(±6.3) | 8.71(±0.30)^#^ | Healthy control | U/S | 8 |
| Rentzos, 2013 | Greece | LN | 36 | 59.2(±10.9) | 10.68(±1.96) | 35 | 56(±11.6) | 11.41(±2.94) | Healthy control | AM | 8 |
| Chen, 2014 | China | LN | 284 | 53.1(±12.1) | 12.04(±2.54) | 217 | 52.71(±14.7) | 11.85(±2.33) | Healthy control | GM, AM | 7 |
| 0ur study, 2020 | China | LN | 245 | 55.9(±8.0) | 11.50(±2.59) | 82 | 55.0(±7.5) | 12.73(±2.74) | Healthy control | GM, AM | 9 |
| Total |  |  | 711 |  |  | 447 |  |  |  |  |  |

^*^Subjects with herniated disc or tension headache with normal CSF findings or clinical and laboratory findings that showed no objective signs of organic neurological diseases; #Values converted into g/L. Abbreviations: RID, radial immunodiffusion; LN, laser nephelometry; U/S, unspecified; AM, age-matched; GM, sex-matched.

**Supplementary Table 2** Studies included in the meta-analysis for evaluating the serum IgA levels between patients with ALS and controls.

| Study | Country | Method of IgA measurement | Cases | | | Control | | | | | NOS score |
| --- | --- | --- | --- | --- | --- | --- | --- | --- | --- | --- | --- |
|  |  |  | n | Mean age (range), years | Mean serum IgG (SD), g/L | n | Mean age (range), years | Mean serum IgG (SD), g/L | Control selection | matching |  |
| Bartfeld,1982 | USA | RID | 43 | U/S | 2.87(±0.22)^#^ | 22 | age range comparable | 2.96(±0.25)^#^ | Spouses and blood donor | GM, AM | 7 |
| Annunziata, 1985 | Italy | RID | 13 | U/S(37-72) | 2.6(±1.16)^#^ | 12 | U/S | 1.79(±0.65)^#^ | Unaffected ^*^ | U/P | 7 |
| Rentzos, 2013 | Greece | LN | 36 | 59.2(±10.9) | 2.38(±1.13)^#^ | 35 | 56(±11.6) | 2.14(±0.89)^#^ | Healthy control | AM | 8 |
| Chen, 2014 | China | LN | 284 | 53.1(±12.1) | 2.10(±0.92) | 217 | 52.71(±14.7) | 2.14(±0.95) | Healthy control | GM, AM | 7 |
| 0ur study, 2020 | China | LN | 245 | 55.9(±8.0) | 2.14(±1.04) | 82 | 55.0(±7.5) | 2.32(±0.98) | Healthy control | GM, AM | 9 |
| Total |  |  | 621 |  |  | 368 |  |  |  |  |  |

^*^Subjects with herniated disc or tension headache with normal CSF findings or clinical and laboratory findings that showed no objective signs of organic neurological diseases; #Values converted into g/L. Abbreviations: RID, radial immunodiffusion; LN, laser nephelometry; U/S, unspecified; AM, age-matched; GM, sex-matched.

**Supplementary Table 3** Studies included in the meta-analysis for evaluating the serum IgM levels between patients with ALS and controls.

| Study | Country | Method of IgM measurement | Cases | | | Control | | | | | NOS score |
| --- | --- | --- | --- | --- | --- | --- | --- | --- | --- | --- | --- |
|  |  |  | n | Mean age (range), years | Mean serum IgG (SD), g/L | n | Mean age (range), years | Mean serum IgG (SD), g/L | Control selection | matching |  |
| Bartfeld,1982 | USA | RID | 43 | U/S | 1.68(±0.17)^#^ | 22 | age range comparable | 1.49(±0.16)^#^ | Spouses and blood donor | GM, AM | 7 |
| Annunziata, 1985 | Italy | RID | 13 | U/S(37-72) | 1.28(±0.47)^#^ | 12 | U/S | 1.64(±0.69)^#^ | Unaffected ^*^ | U/P | 7 |
| Zhang, 2005 | USA | ELISA | 38 | 59.3(±13.4) | 2.28(±2.30)^#^ | 28 | 56.0(±15.1) | 1.37(±1.14)^#^ | Normal blood donor | AM | 8 |
| Saleh, 2009 | USA | U/S | 8 | 53.2(±3.1) | 0.82(±0.13)^#^ | 5 | 59.8(±6.3) | 0.77(±0.24)^#^ | Healthy control | U/S | 8 |
| Rentzos, 2013 | Greece | LN | 36 | 59.2(±10.9) | 0.89(±0.51)^#^ | 35 | 56(±11.6) | 1.05(±0.86)^#^ | Healthy control | AM | 8 |
| Chen, 2014 | China | LN | 284 | 53.1(±12.1) | 1.33(±0.68) | 217 | 52.71(±14.7) | 1.22(±0.60) | Healthy control | GM, AM | 7 |
| 0ur study, 2020 | China | LN | 245 | 55.9(±8.0) | 1.24(±0.59) | 82 | 55.0(±7.5) | 1.12(±0.48) | Healthy control | GM, AM | 9 |
| Total |  |  | 667 |  |  | 401 |  |  |  |  |  |

^*^Subjects with herniated disc or tension headache with normal CSF findings or clinical and laboratory findings that showed no objective signs of organic neurological diseases; #Values converted into g/L. Abbreviations: RID, radial immunodiffusion; LN, laser nephelometry; U/S, unspecified; AM, age-matched; GM, sex-matched.

**Supplementary Table 4** Studies included in the meta-analysis for evaluating the serum C4 levels between patients with ALS and controls.

| Study | Country | Method of C4 measurement | Cases | | | Control | | | | | NOS score |
| --- | --- | --- | --- | --- | --- | --- | --- | --- | --- | --- | --- |
|  |  |  | n | Mean age (range), years | Mean serum IgG (SD), g/L | n | Mean age (range), years | Mean serum IgG (SD), g/L | Control selection | matching |  |
| Annunziata, 1985 | Italy | RID | 13 | U/S(37-72) | 0.51 (±0.22)^#^ | 12 | U/S | 0.53(±0.24)^#^ | Unaffected ^*^ | U/P | 7 |
| Trbojević-Cepe, 1998 | Croatia | LN | 12 | U/S | 0.26(±0.10) | 16 | U/S | 0.26(±0.07) | Unaffected | U/S | 6 |
| Chen, 2014 | China | LN | 284 | 53.1(±12.1) | 0.20(±0.07) | 217 | 52.71(±14.7) | 0.19(±0.05) | Healthy control | GM, AM | 7 |
| 0ur study, 2020 | China | LN | 245 | 55.9(±8.0) | 0.22(±0.06) | 82 | 55.0(±7.5) | 0.21(±0.05) | Healthy control | GM, AM | 9 |
| Total |  |  | 554 |  |  | 327 |  |  |  |  |  |

^*^Subjects with herniated disc or tension headache with normal CSF findings or clinical and laboratory findings that showed no objective signs of organic neurological diseases; #Values converted into g/L. Abbreviations: RID, radial immunodiffusion; LN, laser nephelometry; U/S, unspecified; AM, age-matched; GM, sex-matched.

**Supplementary Table 5** Studies included in the meta-analysis for evaluating the serum C3 levels between patients with ALS and controls.

| Study | Country | Method of C3 measurement | Cases | | | Control | | | | | NOS score |
| --- | --- | --- | --- | --- | --- | --- | --- | --- | --- | --- | --- |
|  |  |  | n | Mean age (range), years | Mean serum IgG (SD), g/L | n | Mean age (range), years | Mean serum IgG (SD), g/L | Control selection | matching |  |
| Bartfeld,1982 | USA | RID | 43 | U/S | 0.9(±0.04)^#^ | 22 | age range comparable | 0.85(±0.04)^#^ | Spouses and blood donor | GM, AM | 7 |
| Chen, 2014 | China | LN | 284 | 53.1(±12.1) | 0.89(±0.20) | 217 | 52.71(±14.7) | 0.86(±0.16) | Healthy control | GM, AM | 7 |
| 0ur study, 2020 | China | LN | 245 | 55.9(±8.0) | 0.85(±0.16) | 82 | 55.0(±7.5) | 0.87(±0.16) | Healthy control | GM, AM | 9 |
| Total |  |  | 572 |  |  | 321 |  |  |  |  |  |

^*^Subjects with herniated disc or tension headache with normal CSF findings or clinical and laboratory findings that showed no objective signs of organic neurological diseases; #Values converted into g/L. Abbreviations: RID, radial immunodiffusion; LN, laser nephelometry; U/S, unspecified; AM, age-matched; GM, sex-matched.
