# Supplementary material for: Evaluation of Peripheral Immune Activation in Amyotrophic Lateral Sclerosis
Source: Front Neurol. 2021 Jun 24;12:628710. doi: 10.3389/fneur.2021.628710 (PMC8264193; doi:10.3389/fneur.2021.628710)
Supplement: Supplementary file 2 [file Data_Sheet_1.PDF]

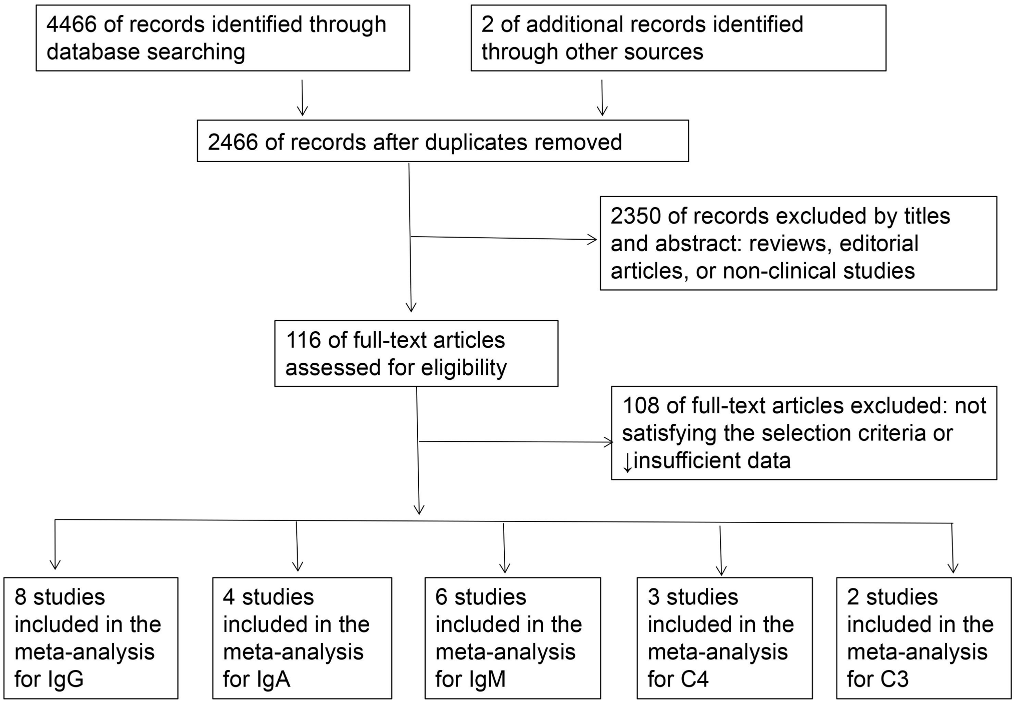

Supplementary Figure 1 Flowchart showing the process of screening studies in the literature for the meta-analysis.

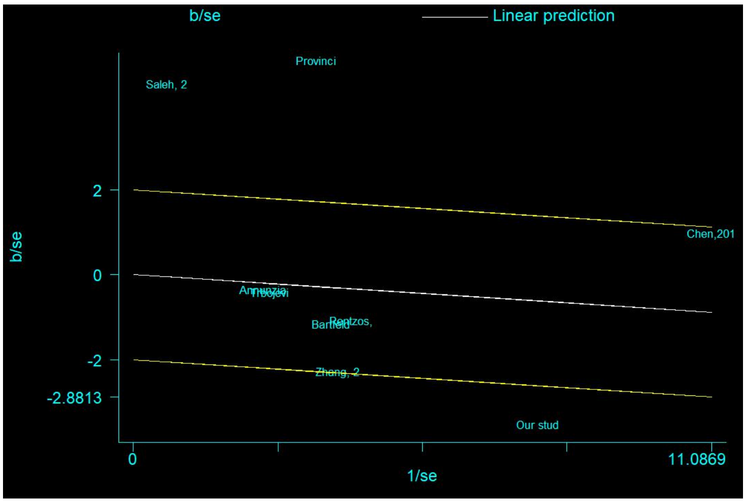

(A)

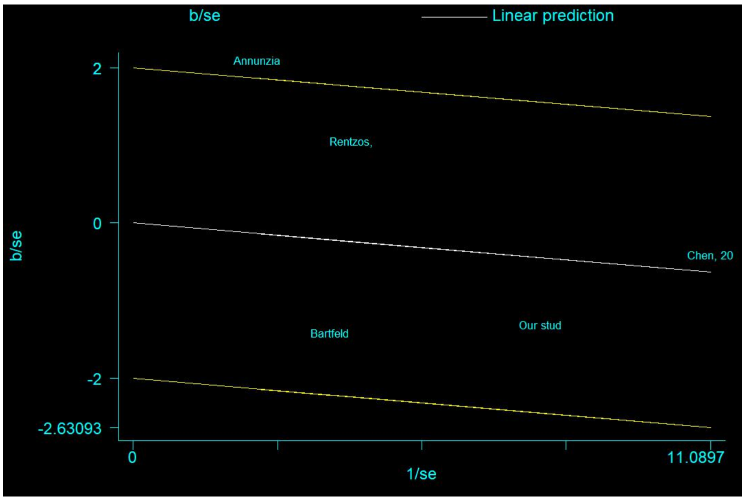

b/se
Linear prediction
Annunzia
Rentzos,
Chen, 20
Our stud
Bartfeld
2
0
-2
-2.63093
b/se
0
11.0897
1/se

(B)

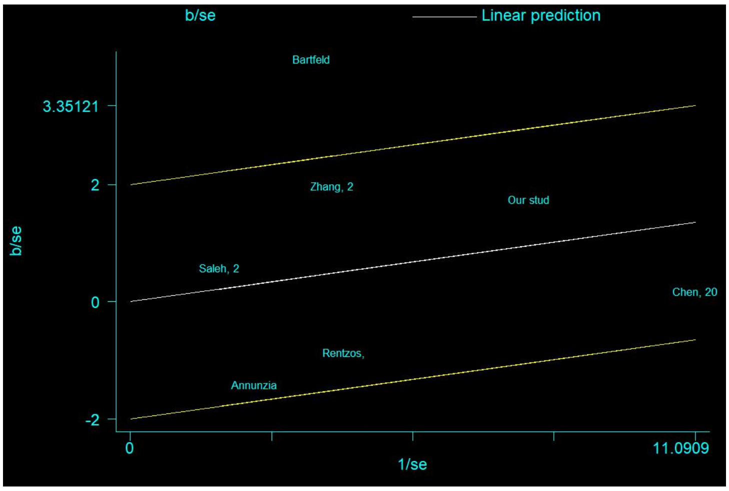

b/se
Linear prediction
Bartfeld
3.35121
2
Zhang, 2
Our stud
Saleh, 2
0
Chen, 20
Rentzos,
Annunzia
-2
b/se
0
11.0909
1/se

(C)

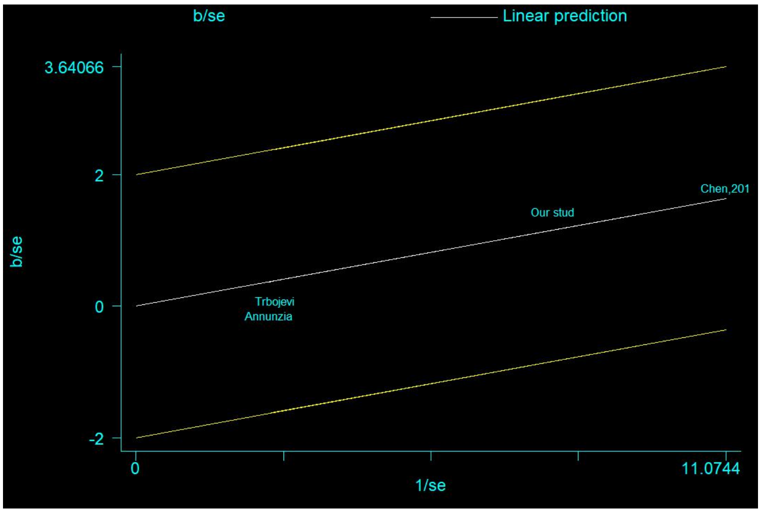

b/se
Linear prediction
3.64066
2
Chen,201
Our stud
0
Trbojevi
Annunzia
-2
b/se
0
11.0744
1/se

(D)

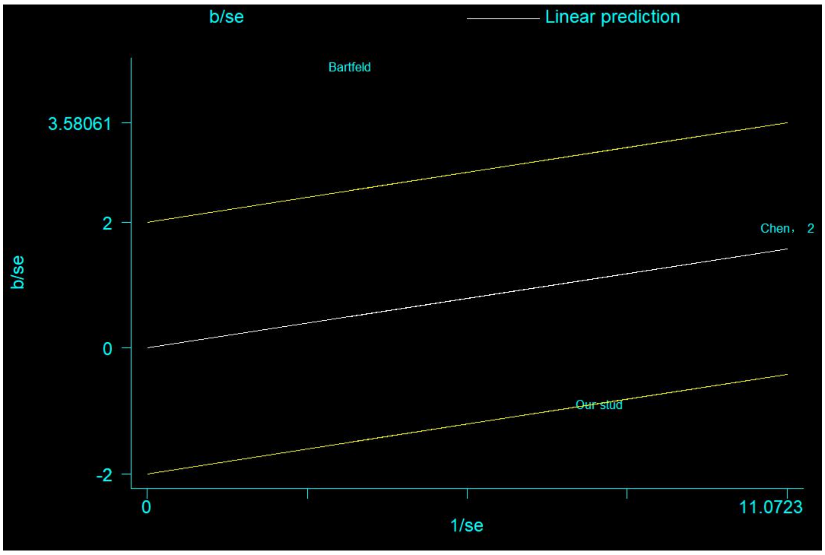

(E)

Supplementary Figure 2 Galbraith graphs for evaluating the heterogeneity across the studies included in the meta-analysis. (A) The heterogeneity across the studies included in the meta-analysis of IgG. Three of nine studies had values exceeding the 95% confidential interval, suggesting substantial heterogeneity. (B) The heterogeneity across the studies included in the meta-analysis of IgA. One of five studies had a value exceeding the 95% confidential interval, suggesting moderate heterogeneity. (C) The heterogeneity across the studies included in the meta-analysis of IgM. One of seven studies had a value exceeding the 95% confidential interval, suggesting moderate heterogeneity. (D) The heterogeneity across the studies included in the meta-analysis of C4. None of four studies had values exceeding the 95% confidential interval, suggesting no heterogeneity. (E) The heterogeneity across the studies included in the meta-analysis of C3. One of three studies had a value exceeding the 95% confidential interval, suggesting substantial heterogeneity.

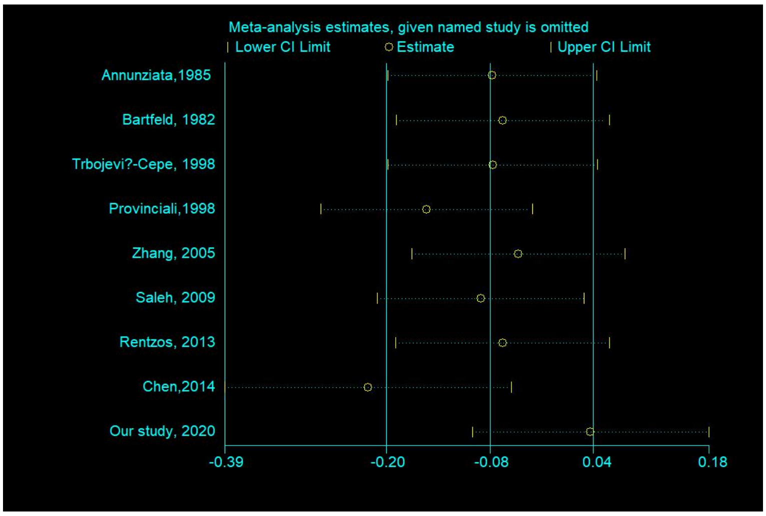
Meta-analysis estimates, given named study is omitted
Lower CI Limit
Estimate
Upper CI Limit
Annunziata,1985
Bartfeld, 1982
Trbojevi?-Cepe, 1998
Provinciali,1998
Zhang, 2005
Saleh, 2009
Rentzos, 2013
Chen,2014
Our study, 2020
-0.39
-0.20
-0.08
0.04
0.18

(A)

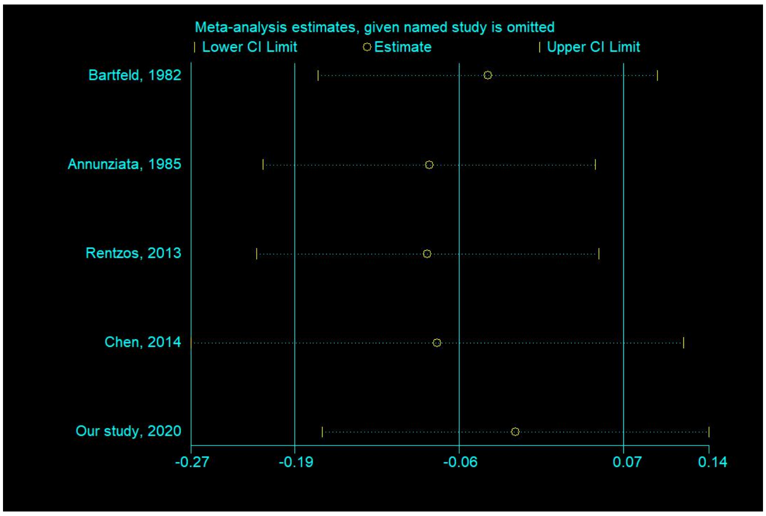
Meta-analysis estimates, given named study is omitted
Lower CI Limit
Estimate
Upper CI Limit
Bartfeld, 1982
Annunziata, 1985
Rentzos, 2013
Chen, 2014
Our study, 2020
-0.27
-0.19
-0.06
0.07
0.14

(B)

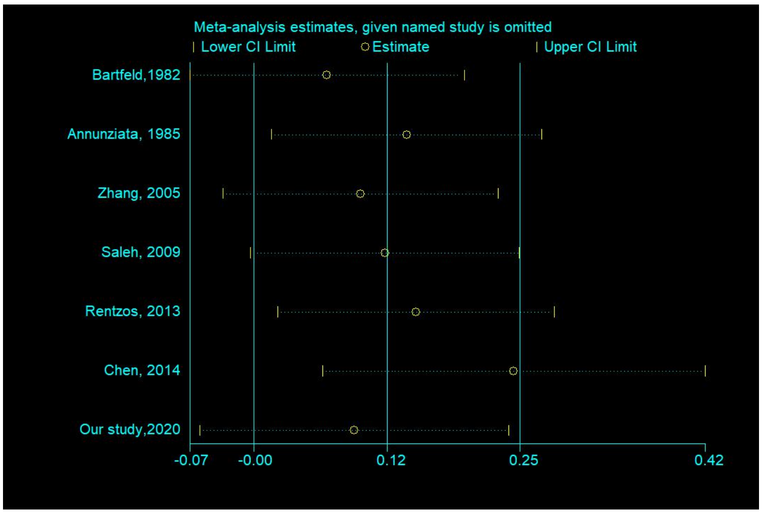
Meta-analysis estimates, given named study is omitted
Lower CI Limit
Estimate
Upper CI Limit
Bartfeld,1982
Annunziata, 1985
Zhang, 2005
Saleh, 2009
Rentzos, 2013
Chen, 2014
Our study,2020
-0.07
-0.00
0.12
0.25
0.42

(C)

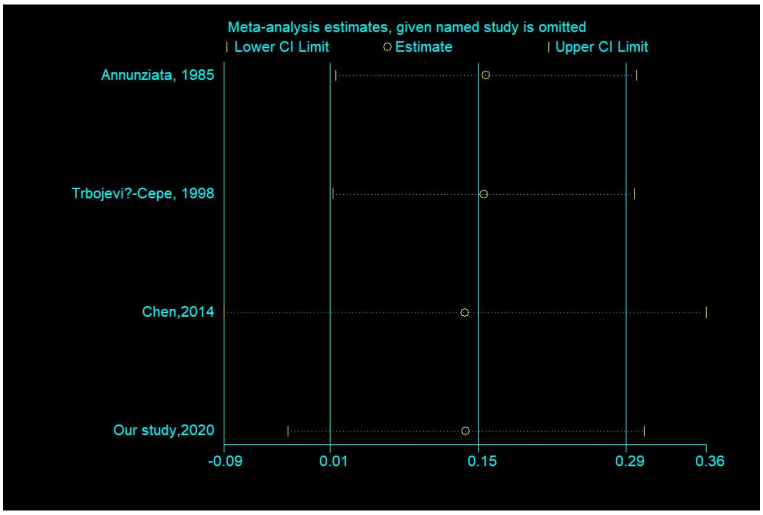

(D)

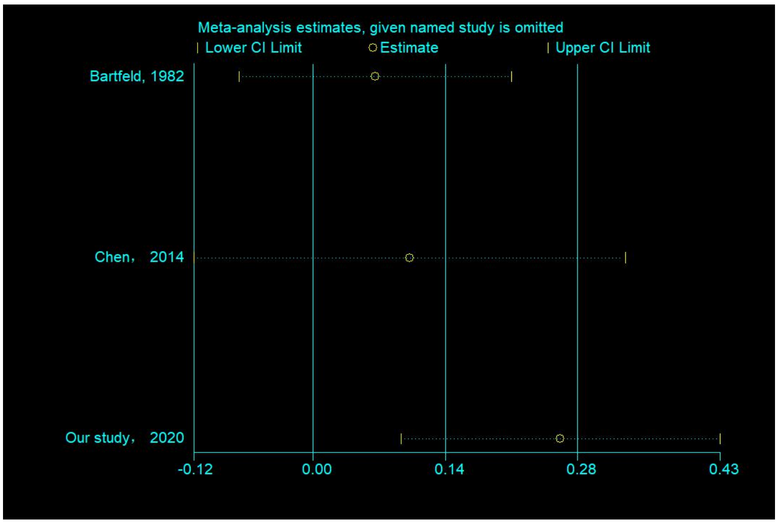

(E)

Supplementary Figure 3 Sensitivity analyses for the meta-analysis performed by study-by-study exclusion. (A) Sensitivity analysis for the meta-analysis of IgG. (B) Sensitivity analysis for the meta-analysis of IgA. (C) Sensitivity analysis for the meta-analysis of IgM. (D) Sensitivity analysis for the meta-analysis of C4. (E) Sensitivity analysis for the meta-analysis of C3.

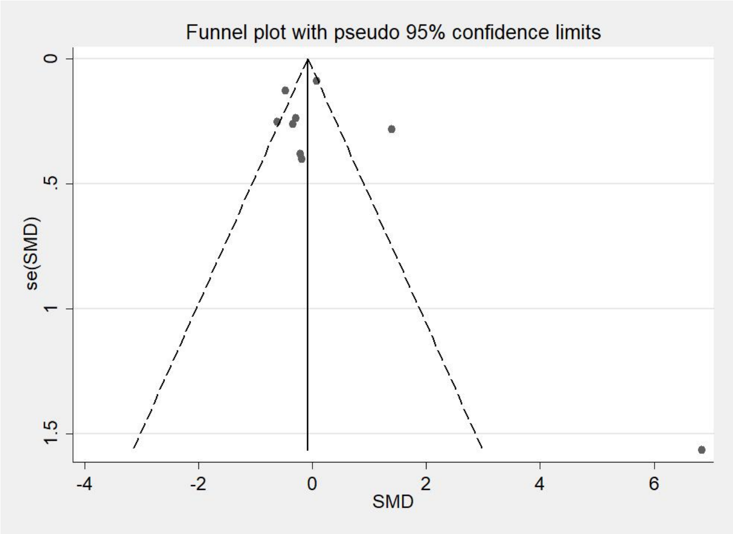
Funnel plot with pseudo 95% confidence limits
se(SMD)
0
.5
1
1.5
-4
-2
0
2
4
6
SMD

(A)

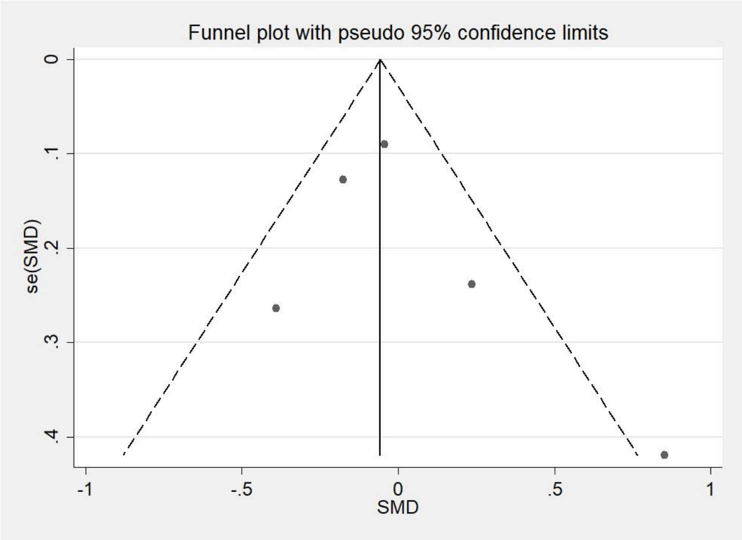
Funnel plot with pseudo 95% confidence limits
se(SMD)
0
.1
.2
.3
.4
-1
-.5
0
.5
1
SMD

(B)

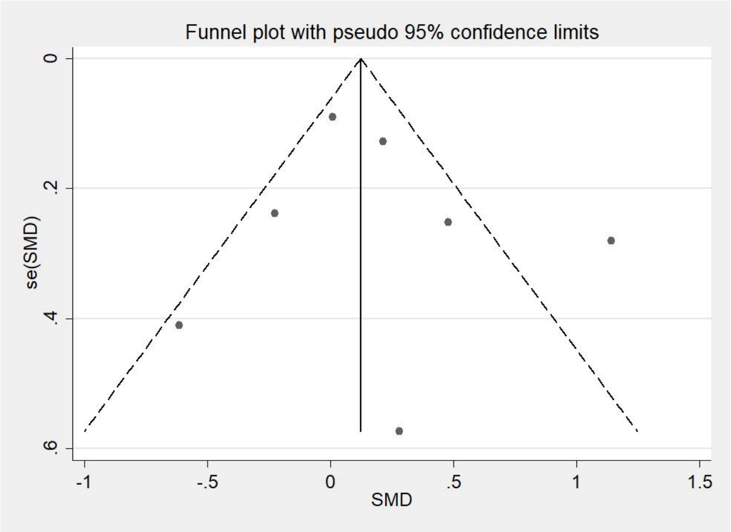
Funnel plot with pseudo 95% confidence limits
se(SMD)
0
.2
.4
.6
-1
-.5
0
.5
1
1.5
SMD

(C)

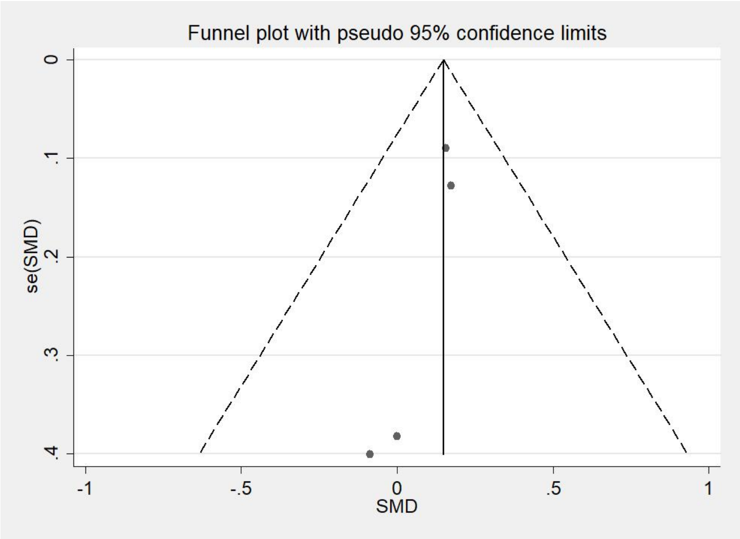

(D)

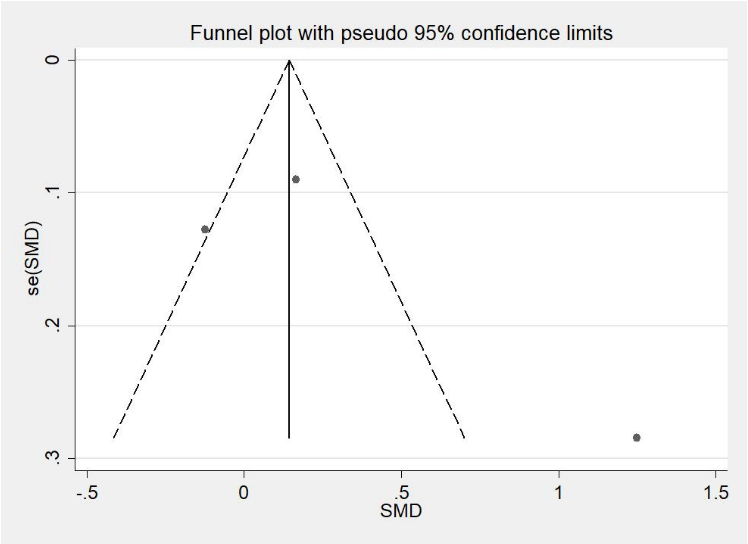

(E)

Supplementary Figure 4 Funnel graph for assessing publication bias. (A) Funnel graph for the meta-analysis of IgG. Egger's and Begg's test results for small study effects: p value=0.455. (B) Funnel graph for the meta-analysis of IgA. Egger's and Begg's test results for small study effects: p value=0.625. (C) Funnel graph for the meta-analysis of IgM. Egger's and Begg's test results for small study effects: p value=0.467. (D) Funnel graph for the meta-analysis of C4. Egger's and Begg's test results for small study effects: p value=0.09. (E) Funnel graph for the meta-analysis of IgG. Egger's and Begg's test results for small study effects: p value=0.97.
